# Learning the facts in medical school is not enough: which factors predict successful application of procedural knowledge in a laboratory setting?

**DOI:** 10.1186/1472-6920-13-28

**Published:** 2013-02-22

**Authors:** Ralf Schmidmaier, Stephan Eiber, Rene Ebersbach, Miriam Schiller, Inga Hege, Matthias Holzer, Martin R Fischer

**Affiliations:** 1 Klinikum der Universität München (LMU), Medizinische Klinik und Poliklinik IV, Ziemssenstr. 1, 80336, Munich, Germany; 2 Lehrstuhl für Didaktik und Ausbildungsforschung (DAM) in der Medizin am Klinikum der Ludwig-Maximilians-Universität München, Munich, Germany

**Keywords:** Conceptual knowledge, Procedural knowledge, Strategic knowledge, Conditional knowledge, Key feature problems, Problem solving task, Clinical experience, Prior cognitive performance

## Abstract

**Background:**

Medical knowledge encompasses both conceptual (facts or “what” information) and procedural knowledge (“how” and “why” information). Conceptual knowledge is known to be an essential prerequisite for clinical problem solving. Primarily, medical students learn from textbooks and often struggle with the process of applying their conceptual knowledge to clinical problems. Recent studies address the question of how to foster the acquisition of procedural knowledge and its application in medical education. However, little is known about the factors which predict performance in procedural knowledge tasks. Which additional factors of the learner predict performance in procedural knowledge?

**Methods:**

Domain specific conceptual knowledge (facts) in clinical nephrology was provided to 80 medical students (3^rd^ to 5^th^ year) using electronic flashcards in a laboratory setting. Learner characteristics were obtained by questionnaires. Procedural knowledge in clinical nephrology was assessed by key feature problems (KFP) and problem solving tasks (PST) reflecting strategic and conditional knowledge, respectively.

**Results:**

Results in procedural knowledge tests (KFP and PST) correlated significantly with each other. In univariate analysis, performance in procedural knowledge (sum of KFP+PST) was significantly correlated with the results in (1) the conceptual knowledge test (CKT), (2) the intended future career as hospital based doctor, (3) the duration of clinical clerkships, and (4) the results in the written German National Medical Examination Part I on preclinical subjects (NME-I). After multiple regression analysis only clinical clerkship experience and NME-I performance remained independent influencing factors.

**Conclusions:**

Performance in procedural knowledge tests seems independent from the degree of domain specific conceptual knowledge above a certain level. Procedural knowledge may be fostered by clinical experience. More attention should be paid to the interplay of individual clinical clerkship experiences and structured teaching of procedural knowledge and its assessment in medical education curricula.

## Background

Medical school should prepare students for their first work day as a physician. One of the major learning objectives in medical education is the ability to solve clinical problems by application of learned knowledge to an individual patient case. Conceptual knowledge as knowledge about the declarative textbook facts is of significant importance for problem solving [1-3]. Procedural knowledge of solving medical problems is different from conceptual knowledge one possesses about the medical problem [4]. Learners need to understand the problem to be capable of solving problems from novel categories [5]. Understanding in this context means knowledge of the domain and of its teleology [6]. Strategic knowledge comprises knowledge about problem-solving strategies and heuristics in the process [7], whereas conditional knowledge is knowledge about the conditions of application of conceptual and strategic knowledge which also implies knowledge about the rationale behind the selection of diagnostic or therapeutic decisions [7]. Interestingly, reasoning experts struggle with solving problems outside their own domains underlining the need for sufficient domain knowledge [8-12].

### Model for clinical knowledge

**Figure 1:**
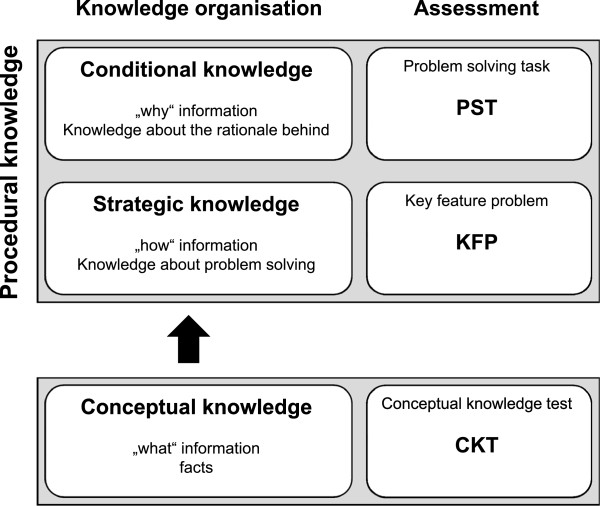
**Model of diagnostic knowledge and assessment according to van Gog **[5]**and Kopp **[7,13]**.**

Unfortunately the nomenclature of clinical knowledge is not uniform. We adopted a model in which clinical knowledge is comprised of conceptual knowledge (facts, “what” information), strategic knowledge (“how” information), and conditional knowledge (“why” information) [7,13]. Conceptual knowledge means declarative textbook facts, whereas strategic knowledge and conditional knowledge constitute procedural knowledge (knowledge organization is shown in Figure 1).

### Assessment of clinical knowledge

**Figure 2:**
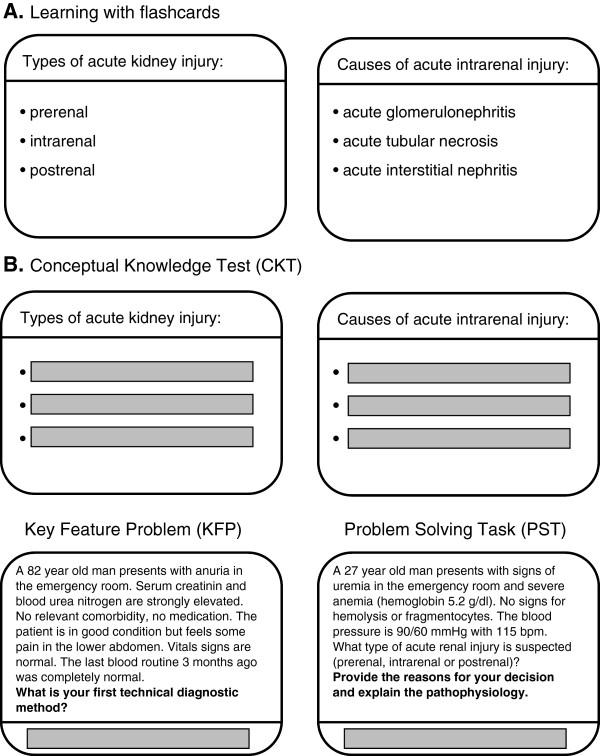
Learning (A) and Assessment (B) Tools.

Conceptual knowledge can be acquired and assessed by flashcards [14-16]. We used this approach for our conceptual knowledge test (CKT, Figure 2). Key feature problems (KFP) were used to measure strategic knowledge while problem-solving tasks (PST) were used to assess conditional knowledge [17-22]. A key feature problem consists of a clinical case scenario followed by questions that focus on only the critical steps in the resolution of the clinical problem [23]. A problem-solving task (PST) consists of a clinical case scenario followed by making a clinical decision. Participants are asked to explain their decision and to describe the underlying pathophysiological process [7,13].

### Teaching procedural knowledge

The ability to solve clinical problems and to find the right diagnosis is a core task in medicine, but the knowledge about how to teach procedural knowledge is still limited [24]. It is known from the literature that students struggle to find the right diagnosis although they possess the required conceptual knowledge [25]. Process approaches, in contrast to pure product approaches, have been proven to be effective instructional strategies to foster the application of conceptual knowledge They seek to attain transfer by having novices mimic experts’ problem-solving behaviour during training [5]. Further, it has been shown that clinical problems can also be approached by non-analytic reasoning which is based on clinical experience [26]. Despite these promising approaches, there is still a lack of knowledge about why students struggle to apply the acquired knowledge [25]. One explanation could be that the content alone is not sufficient for successful application but needs to be structured or organized [27]. Little is known about how to advance the organization of conceptual knowledge and which factors influence this organization process. A better understanding of conceptual knowledge organization and its impact on procedural knowledge application to clinical problems would allow for new teaching approaches. Ultimately this teaching could foster the ability to solve clinical problems and thus reduce the rather high number of wrong diagnoses in medicine [28].

### Potential factors predicting performance in procedural knowledge

The aim of our study was to identify factors, in addition to domain specific conceptual knowledge, that predict superior performance in procedural knowledge tasks. For this we collected data on learner characteristics that may predict performance in medical school [29,30]. Motivation, learning style, personal goals, gender and age were revealed by questionnaires. Additionally clinical experience may be associated with better procedural knowledge. In German medical schools the students earn the majority of their clinical experience in their final year (6^th^ year or practical year). Prior to this final year the individual experience is limited to four clinical clerkships of one month duration which have to be done in the 3^rd^, 4^th^ or 5^th^ year in addition to the structured clinical curriculum. We used the duration of these clerkships as surrogate marker for individual clinical experience.

After two years of medical school, German students have to take the written National Medical Examination Part I on preclinical subjects (NME-I). It includes 320 multiple choice questions which are nationwide uniform and assesses the conceptual knowledge mainly in anatomy, biochemistry and physiology but also in psychology, biology, chemistry and physics. In the field of medical education NME-I performance and university entrance diploma (“Abitur”) were reported as reliable measures of prior cognitive performance in Germany [31-33].

### Aim of study

To learn more about the factors of the learners which are correlated with superior procedural performance we conducted a controlled laboratory study including 80 medical students. Several learner characteristics such as clinical clerkship experience, prior cognitive performance or other individual characteristics were obtained by questionnaires.

As conceptual knowledge is a prerequisite and a known strong influencing factor [1-3], this variable was controlled by an intensive, standardized, uniform conceptual knowledge training using electronic flashcards within the domain of clinical nephrology. Performance in KFP and PST were defined as dependent variables to assess procedural knowledge in medical education.

## Methods

### Participants

80 students from Ludwig-Maximilians-University (LMU) in Munich participated voluntarily in the study (34 males, 46 females). All participants were in the clinical part of the medical curriculum (3^rd^ to 5^th^ year). The participating students were representative for our medical school in terms of the available characteristics age (23.7 vs. 23.4 years; P=0.48), university entrance diploma (1.71 vs. 1.62; P=0.25) and self-reported NME-I result (2.58 vs. 2.55; P=0.82) (see Additional file 1). Students signed an informed consent form and received compensation for expenses. The ethical committee of the University of Munich approved the study. There are no conflicts of interest.

### Study design

The study consisted of a learning phase directly followed by an assessment phase and was carried out in a laboratory setting. All materials were evaluated during a pilot phase with twelve participants and refined accordingly. During the learning phase all participants were exposed to 30 electronic flashcards four times in a row to uniformly provide conceptual knowledge of clinical nephrology (Figure 2A). The participants studied in a self-directed manner. The flashcards included a title (cue) and learning objectives (targets) and were displayed on one screen of the CASUS™ system [34,35]. There was no time limitation in the learning phase with an average time need of 120 minutes. At the end of the learning phase participants were able to reproduce a mean of 85.9% (95% confidence interval ± 2.5%) of all flashcard items (targets) completely correct when the cues were given. After that optical illusions called ambiguous images were used as a cognitive distractor task in order to close up the learning phase and to change the focus before beginning of the assessment. In the subsequent assessment phase participants were tested for their conceptual and procedural knowledge. For conceptual knowledge test (CKT) participants had to actively recall the 30 flash card items when the cues were given (CKT, Figure 2B). Then the participants had to solve three key feature problems (KFP) each consisting of five items, and had to work on three problem solving tasks (PST) each consisting of five items. Participants did not receive any feedback to their performance. No time limitation was given in the assessment phase. Figure 2C shows examples for a KFP and a PST. All responses were recorded electronically. Flashcards, KFP and PST were developed by medical experts and experienced educators. The cases were derived from real clinical encounters. The clinical cases of KFP and PST were strongly linked to the flashcard contents (see Figure 2 and Additional file 2). For rating KFPs and PSTs standardized checklists were applied by two independent raters. All cases of divergent ratings were discussed and jointly decided on. The co-variables were acquired via questionnaires. All participants filled out a questionnaire regarding their psycho-social background, gender, age, motivation, mood, frequently used learning strategies, prior knowledge, diploma performance, percentage of correct answers in NME-I, and intended future career. Additionally, the learning style of each participant was determined with the Kolb’s Learning Style Inventory [36].

### Statistics

Association of procedural knowledge with other covariables was analyzed by correlation studies and multiple regression analysis. P values were calculated for group comparisons of normally distributed values with Student’s t-test, for correlative relationships with the Pearson product–moment correlation and for categorical independent variables with ANOVA. P values < 0.05 were considered as statistically significant. All tests were calculated using the GraphPad Prism software or the R software (R version 2.11.1).

## Results

### Available conceptual knowledge test in clinical nephrology

**Figure 3:**
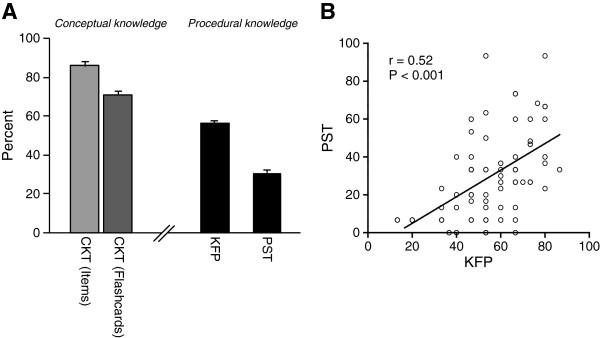
(A) Performance in CKT, KFP and PST (B) and correlation between procedural knowledge tests.

Assessing conceptual knowledge by CKT confirmed excellent domain specific knowledge of the participants: 85.9% of single flashcard items were recalled correctly. Regarding entire flashcards and not single items, 71.0% flashcards were recalled completely correct (Figure 3A).

### Achievement in key feature problems (KFP) and problem-solving tasks (PST) for the assessment of procedural knowledge

In contrast to CKT, the participants struggled with procedural knowledge tasks. Only 56.2% percent of the leading diagnoses and diagnostic steps were correctly identified in the three KFPs reflecting strategic knowledge. The results in the PSTs testing for conditional knowledge were even worse. The participants explained their decisions and described the pathophysiological processes in 30.4% of items correctly (Figure 3A).

The overall performance in both procedural knowledge tests (sum of KFP and PST) was weakly correlated with the performance in conceptual knowledge test (CKT) (r(sum of KFP+PST / CKT)=0.27; P=0.017). However, the performances in both procedural knowledge tests, namely KFP and PST, were strongly correlated with each other (r(KFP / PST)=0.52; P<0.001), suggesting that both tests measure similar cognitive abilities (Figure 3B).

### Correlation of learner characteristics with performance in procedural knowledge tests

Table 1 shows that age, years of study, gender, and learning style are not significantly correlated with procedural knowledge. Self-assessment of motivation to learn nephrology showed significant correlation only with KFP performance (P=0.040), but not with PST and with the combination of KFP and PST. We asked whether the participants prefer to work as a general practitioner, as a hospital-based doctor, or whether they aspire to an academic career in the future. Interestingly students intending hospital-based work showed the best performance in procedural knowledge tests. Students who want to work as general practitioners struggled most in these tests. The duration of clinical clerkships as an indicator for clinical experience of students significantly correlates with their performance in KFPs (r=0.33; P=0.003) but not in PSTs (r=0.14, P=0.218). Prior cognitive performance in medical school seems to be the best predictor for all kinds of knowledge in our setting: NME-I performance is highly correlated with performance in CKT (P=0.004), KFP (P=0.025), PST (P=0.001), and overall procedural knowledge (sum of KFP+PST; P=0.001). The results of university entrance diploma as a parameter for prior cognitive performance in non-medical domains is not significantly correlated with either KFP or with PST.

**Table 1:** Correlations between performance in procedural knowledge tests (KFP and PST) and learner characteristics

	**Procedural knowledge (KFP + PST)**		**Strategic knowledge (KFP)**		**Conditional knowledge (PST)**	
	**r**	**P**	**r**	**P**	**r**	**P**
**Domain specific conceptual knowledge**						
Result of conceptual knowledge test	0.27	0.017*	0.18	0.100	0.26	0.019*
**Learner characteristics**						
Age	0.09	0.444	0.13	0.266	0.04	0.740
Gender, F/M		0.190		0.141		0.380
Kolb’s Learning Style Inventory		0.361		0.942		0.154
Motivation to learn nephrology	0.12	0.287	0.23	0.040*	0.01	0.911
Intended career as hospital-based doctor		0.009**		0.009**		0.068
**Clinical experience**						
Years of study	0.07	0.561	0.12	0.305	0.02	0.895
Duration of clinical clerkships	0.26	0.023*	0.33	0.003**	0.14	0.218
**Cognitive performance**						
Result of university entrance diploma	−0.07	0.541	0.11	0.380	−0.18	0.130
Percentage of correct answers in NME-I	0.38	0.001**	0.26	0.025*	0.38	0.001**

* p < 0.05; ** p < 0.01; *** p < 0.001.

### Clinical experience and cognitive performance in medical school influence performance in procedural knowledge tests

As there are multiple interrelations between the above analyzed parameters such as motivation, intended future career, and prior cognitive performance, we ran a multiple regression analysis to find independent factors that significantly influence performance in procedural knowledge. Due to the negative results in single correlations, age, gender and years of study were omitted from this analysis. Table 2 shows that only prior cognitive performance in medical school (“NME-I performance”) and clinical experience (“duration of clinical clerkships”) remain significant and independent factors.

**Table 2:** Multiple regression analysis for independent factors that influence procedural knowledge performance (KFP and PST)

	**Procedural knowledge (KFP + PST)**		**Strategic knowledge (KFP)**	**Conditional knowledge (PST)**
	**P**	**β**	**P**	**P**
**Domain specific conceptual knowledge**				
Result of conceptual knowledge test	0.150	0.238	0.146	0.267
**Learner characteristics**				
Kolb’s Learning Style Inventory	0.667		0.617	0.772
Motivation to learn nephrology	0.226	−0.176	0.999	0.092
Intended career as hospital-based doctor	0.133		0.243	0.168
**Clinical experience**				
Duration of clinical clerkships	0.022* (r^**2**^**=0.053)**	**0.315**	0.0002*** (r^**2**^**=0.096)**	0.466
**Cognitive performance**				
Result of university entrance diploma	0.714	0.068	0.174	0.725
Percentage of correct answers in NME-I	0.025* (r^**2**^**=0.136)**	**0.376**	0.010* (r^**2**^**=0.055)**	0.122
**Adjusted r**^**2**^	0.2621		0.3395	0.1497

* p < 0.05; ** p < 0.01; *** p < 0.001.

## Discussion

### Relation of conceptual knowledge to procedural knowledge in medical school

Building on past studies [7,13,37], the several dimensions of clinical knowledge were assessed by CKT, KFP, and PST. The participants performed well in the CKT, which emphasizes that flashcards are a practicable and efficient way to acquire conceptual (factual) knowledge in a controlled laboratory setting [14,15].

Conceptual knowledge is thought to be a prerequisite for clinical decisions. However, in our multiple regression analysis the performance in CKT was not a significant independent factor. This means that after giving a standardized learning phase for conceptual knowledge, good performers and poor performers do not differ significantly in their ability to make clinical decisions (KFP) and to solve clinical problems (PST).

The results in the procedural knowledge tests (KFP and PST) showed a strong correlation (Figure 3, r=0.52; P < 0.001) with each other indicating that these two test types appear to assess a similar competency in the process of application of conceptual knowledge to clinical cases, which is believed to be a fundamental task of physicians and called procedural knowledge in our study.

The participants scored surprisingly low in the procedural knowledge tasks of the same content domain. Our interpretation is that learning by flashcards promotes predominantly conceptual knowledge rather than other kinds of knowledge needed for clinical work such as strategic or conditional knowledge [7,13,37]. One consideration is that these tests assess different cognitive abilities and therefore a standard level of difficulty cannot be set. Although multiple item reviews and a pilot trial had been conducted in advance, we cannot exclude that KFP and PST items were more difficult in comparison to the CKT items. Analysing the presented data, it can only be inferred and not proven that our sample of medical students have problems applying conceptual knowledge to clinical cases as stated by other authors [5,25]. As the participants were obviously equipped with the needed conceptual knowledge, it is possible that a lack of organization of this knowledge [27] caused the inability to transfer the knowledge on the procedural knowledge tests. Notably we did not test for transfer as the act of applying conceptual knowledge learned in one context to solve a problem in a novel context [38,39] but for the ability to apply conceptual knowledge to a higher knowledge dimension within the same content domain of clinical nephrology [4].

### Factors associated with superior procedural knowledge

Age and gender did not influence the results of procedural knowledge tests. The learning style, as assessed by the Kolb’s Learning Style Inventory was also not correlated with procedural knowledge. Procedural knowledge did not increase with the years of study, contrasting to former findings [40]. This may be due to the fact that all participants went through the same content related learning phase.

Motivation to learn nephrology was significantly correlated with performance in KFP. It is known that motivation can significantly influence the learning process [41,42] and does correlate with study achievements [41,43,44]. However, in our study self-assessed motivation to learn was not an independent factor.

We also asked the participants about their career plans. Future general practitioners showed the poorest performance in KFP and PST. Moreover future hospital-based doctors showed better results than future academics suggesting that the ability to apply conceptual knowledge is perhaps influenced by the concrete motivation for clinical work – or vice versa. These results are difficult to interpret as some studies showed a relationship between study achievements and intended career [45] but others did not [46,47].

NME-I performance is a strong and independent predictor for performance in procedural knowledge (KFP+PST). NME-I consists of defined number of MCQ for every preclinical science like anatomy, biochemistry or physiology. No knowledge transfer between the subjects is required and no clinical cases are included in NME-I. Therefore we assume that NME-I mainly measures basic biomedical conceptual knowledge. As there is only a minimal if any overlap between these learning objectives and the conceptual knowledge in our study we can only presume what causes the strong correlation with the performance in procedural knowledge (KFP+PST). We think that students with an outstanding performance in NME-I have a better structure and organization of the conceptual knowledge and a better understanding of basic scientific principles and processes. This might help to organize new facts in a more efficient way leading to a better application to procedural knowledge tasks.

Of note, an earlier and more general parameter for prior cognitive performance (university entrance diploma) did not correlate with procedural knowledge.

The second independent factor for procedural knowledge in our study was the duration of clinical clerkships suggesting that the exposure to real life clinical settings might foster procedural knowledge itself. The mechanisms of this process need to be elucidated for example by learning diaries [48].

Of note, years of study was not an influencing factor, although clinical experience was. This was surprising given that clinical experience probably correlates with years of study. However, German medical students can freely choose in which year they do their clinical electives resulting in some variability which might contribute to the negative finding for years of study.

### Limitations of the study

This is a prospective controlled laboratory study with 80 3^rd^ to 5^th^ year students. The used assessment tests for conceptual and procedural knowledge have proven effective in studies with similar sample sizes [7,13,16]. As stated above the performance in the procedural knowledge tests may be underestimated due to high items difficulty. Additionally only one domain (clinical nephrology) was tested in an electronic environment at one medical school thus limiting generalizability and ecological validity of results. As this study analyses only correlations, interventional studies need to be done.

### Future perspectives

The process of applying the conceptual knowledge to the clinical setting needs to be further analyzed to better understand which cognitive strategies are helpful for effective training of procedural knowledge in medical school. Upcoming studies should address effects on knowledge retention, should be conducted in a multicenter design as well as in an ecologically more valid setting. Furthermore the mechanisms of the established influencing factors are not understood. Maybe cognitive organization of the different concepts needs to be explained and fostered.

We conclude that assessment in medical school must not be reduced to CKT such as multiple-choice questions since good performance in CKT does not predict the ability to apply this knowledge to clinical cases. Key feature problems are a well-established approach for testing clinical decision making [17,23] which has proven effective in different settings [18-21] and can also be used via online platforms [22]. Creating key feature problems is an easy process to learn and the assessment of them requires only limited effort [20]. In case of shortage of time or resources we recommend key feature problems instead of problem solving tasks for evaluation of procedural knowledge in medical schools. Another interesting question for further research is to which extent clinical training with real patients should be preceded by training with virtual or simulated patients [49].

## Conclusions

The study offers a proof of concept for the construct and assessment of procedural knowledge. Prior medical school achievements and clinical experience are strong and independent factors affecting performance in the procedural knowledge assessment. Educators should better understand how working with clinical cases and how practical experience foster procedural knowledge most efficiently. In order to improve procedural knowledge in medical students, the medical curriculum should emphasize elements which enhance clinical experience [50].

### Practice points

Conceptual knowledge is not sufficient for the successful application of procedural knowledge. Procedural knowledge is influenced by prior cognitive performance in medical school and by clinical experience.

## Competing interests

The authors declare that they have no competing interests.

## Authors’ contributions

RS was the principal investigator. RS formulated and designed the study, conducted the experiment, collected and analyzed the data and wrote the first draft of the manuscript. SE analyzed the data, helped to conduct the experiment and helped to draft the manuscript. RE helped to design the study, was substantially involved in the planning and the realization of the laboratory experiment and the acquisition of the data. MS helped to conduct the laboratory experiment and substantially contributed to the collection and the processing of the data. IH realized the electronic instruction and assessment tool. MH helped to design the study, was involved in the planning and the conduction of the experiment, helped to realize the electronic learning and assessment tool, helped to analyze the data and performed the statistical analysis. MF was the mentor of the project and was involved in all steps of the project; he substantially contributed to the design of the study and the interpretation of the data and to the manuscript. All authors read and approved the final manuscript.

## Pre-publication history

The pre-publication history for this paper can be accessed here:

http://www.biomedcentral.com/1472-6920/13/28/prepub

## Supplementary Material

Additional file 1: Table S1 Connection between flashcard contents and the procedural tasks. Click here for file

Additional file 2: Table S2 Characteristics of the study group. Click here for file
