# Supplementary material for: Learning the facts in medical school is not enough: which factors predict successful application of procedural knowledge in a laboratory setting?
Source: BMC Med Educ. 2013 Feb 22;13:28. doi: 10.1186/1472-6920-13-28 (PMC3598785; doi:10.1186/1472-6920-13-28)
Supplement: Additional file 1: Table S1 — Connection between flashcard contents and the procedural tasks. [file 1472-6920-13-28-S1.pdf]

Supplemental Table 1.

Connection between flashcard contents and the procedural tasks.

concepts	flash cards																													
	1	2	3	4	5	6	7	8	9	10	11	12	13	14	15	16	17	18	19	20	21	22	23	24	25	26	27	28	29	30
normal kidney function	X	X				X		X	X	X	X	X	X																	
pathological kidney function	X	X		X		X	X	X	X	X	X	X	X	X		X	X	X	X	X		X				X	X			X
renal diseases				X	X		X							X	X	X	X	X	X	X	X	X	X	X	X	X	X	X	X	X
clinical presentation			X	X			X				X	X	X	X	X	X				X	X		X	X	X	X	X	X	X	X
**procedural tasks**																														
PST-1	X	X	X	X	X	X	X	X	X	X		X	X	X	X	X	X	X	X	X	X			X				X		X
PST-2	X		X	X	X		X	X	X	X	X							X	X						X	X	X			
PST-3	X	X					X							X			X					X		X					X	X
KFP-1	X	X	X	X	X		X					X	X		X	X		X	X	X	X		X		X	X	X			
KFP-2	X	X	X	X	X	X	X	X		X		X	X							X				X				X	X	X
KFP-3		X	X	X	X	X	X					X	X	X	X	X	X	X	X		X	X	X	X	X	X	X		X	X
