# Supplementary material for: Learning the facts in medical school is not enough: which factors predict successful application of procedural knowledge in a laboratory setting?
Source: BMC Med Educ. 2013 Feb 22;13:28. doi: 10.1186/1472-6920-13-28 (PMC3598785; doi:10.1186/1472-6920-13-28)
Supplement: Additional file 2: Table S2 — Characteristics of the study group. [file 1472-6920-13-28-S2.pdf]

**Supplemental Table 2.**

**Characteristics of the study group.**

| | Study group | Medical school | P |
|---|---|---|---|
| **3rd year students** (number) | 19 | 481 | |
| **4th year students** (number) | 30 | 451 | |
| **5th year students** (number) | 31 | 465 | |
| **Age** (mean of 3rd year students) | 23.7 | 23.4 | 0.483 |
| **University entrance diploma** (mean) | 1.71 | 1.62 | 0.251 |
| **NME-I result** (mean) | 2.58 | 2.55 | 0.820 |
